# Perioperative enriched environment attenuates postoperative cognitive dysfunction by upregulating microglia TREM2 via PI3K/Akt pathway in mouse model of ischemic stroke

**DOI:** 10.3389/fnins.2024.1520710

**Published:** 2024-12-20

**Authors:** Yuchen Yao, Liru Hu, Danni Li, Yuhao Wang, Jian Pan, Dan Fan

**Affiliations:** ^1^Department of Anesthesiology, Sichuan Provincial People’s Hospital, School of Medicine, University of Electronic Science and Technology of China, Chengdu, China; ^2^State Key Laboratory of Oral Diseases, Department of Oral and Maxillofacial Surgery, National Center for Stomatology and National Clinical Research Center for Oral Diseases, West China Hospital of Stomatology, Sichuan University, Chengdu, China

**Keywords:** enriched environment, POCD, ischemic stroke, microglia, TREM2

## Abstract

Postoperative cognitive dysfunction (POCD) is a prevalent complication that significantly affects the quality of life. Notably, patients who have experienced ischemic stroke are at an increased risk of developing POCD. Exploring the underlying mechanisms of POCD is crucial for its management. Numerous studies have established neuroinflammation as an independent risk factor in POCD pathogenesis, with TREM2 emerging as a key neuroprotective factor that modulates neuroinflammatory responses through the PI3K/Akt signaling pathway. In this study, we aimed to investigate the effect of TREM2 on POCD in a mouse model of ischemic stroke, with a focus on the mechanisms involving TREM2 and the PI3K/Akt signaling pathway. Our findings indicated that mice with ischemic stroke exhibited severe cognitive impairment after surgical trauma. However, we observed that an enriched environment (EE) could ameliorate this cognitive impairment by upregulating microglia TREM2 expression in the hippocampus and suppressing neuroinflammation. Additionally, the PI3K/AKT signaling pathway was activated in the hippocampal tissue of the mice housed in EE. Importantly, the beneficial neuroprotective and anti-inflammatory effects of EE were abolished when TREM2 was knocked down, underscoring the essential role of TREM2 in mediating the effects of EE on neuroinflammation and cognitive function after ischemic stroke and surgical trauma. In general, our study has confirmed a potential molecular mechanism that led to the occurrence of POCD in individuals with ischemic stroke and provided new strategies to treat POCD.

## Introduction

Ischemic stroke, characterized by a sudden occurrence of focal neurological deficits due to reduced blood supply to a particular region of the brain, is intimately linked to cognitive impairment, memory deterioration, and dementia. This condition places a substantial strain on global public health systems (Mozaffarian et al., 2015; GBD 2019 Stroke Collaborators, 2021). Against the backdrop of a growing aging population, the incidence of ischemic stroke among patients in the perioperative period is on the rise annually. These patients are particularly vulnerable to postoperative cognitive dysfunction (POCD), which significantly hampers postoperative recovery and diminishes the quality of life (NeuroVISION Investigators, 2019; Messé et al., 2024).

The pathophysiological changes associated with the combination of POCD and ischemic stroke are intricate, encompassing neuroinflammation, blood–brain barrier disruption, epigenetic modifications, amyloid-beta accumulation, and excessive tau phosphorylation (He et al., 2024). Although considerable research has been conducted, the precise mechanisms underlying stroke-induced damage remain not entirely elucidated. Normal brain function is critically reliant on a steady supply of oxygen and nutrients, and proinflammatory cytokines unleashed during ischemic and anoxic episodes can trigger neuroinflammation, which may be localized or systemic, leading to neuronal damage in the hippocampus (Zhang et al., 2016, 2018). Consequently, neuroinflammation within the central nervous system (CNS) remains a paramount mechanism that merits further investigation for POCD in patients who have experienced ischemic stroke.

Microglia, critical immune cells within the CNS, are instrumental in preserving brain homeostasis through the mediation of immune responses, phagocytosis of pathogens, and the modulation of neuronal activity (Borst et al., 2021). A study by Thiel A et al. noted a significant activation of microglia following ischemic stroke in mice, which triggers chronic CNS inflammation and subsequent neuronal damage, particularly in the hippocampal dentate gyrus region (Thiel et al., 2010). Additionally, general anesthesia and surgical trauma have been shown to disrupt the homeostasis of CNS and exacerbate cognitive impairment by affecting microglial function (Jin et al., 2023; Sheu et al., 2023). TREM2 is an immune regulatory receptor predominantly found in microglia. It associates with DAP12 to form transmembrane receptor signaling complexes, which play a role in anti-inflammatory processes (Wang et al., 2015, 2022). Research has indicated that TREM2 deficiency can severely compromise microglial activation, inflammatory response, and phagocytic capabilities. Moreover, the absence of TREM2 has been associated with increased infarct size and hindered neurological recovery in mice post-ischemia due to reduced microglial activation (Cantoni et al., 2015). These findings underscore the pivotal role of TREM2 in the regulation of brain tissue repair and the recovery of neurological function, highlighting its significance as a key receptor in microglia.

The PI3K/Akt signaling pathway is integral to various cellular functions, including the regulation of neurogenesis, synaptic plasticity, and neuronal metabolism within the nervous system (Chen et al., 2022; Vasconcelos de Matos et al., 2023). TREM2 has been identified to play a neuroprotective role by engaging the PI3K/Akt signaling pathway to regulate neuroinflammatory responses (Wang et al., 2020). Besides, this regulatory mechanism is crucial for maintaining CNS homeostasis and promoting recovery after neurological insults following ischemic injury (Chen et al., 2020).

The concept of an enriched environment (EE) was first introduced in 1947, defining it as a living space designed with cognitive, physical, and social stimuli, in contrast to a standard environment (SE) (Alwis and Rajan, 2014). Research by Wang et al. has demonstrated that 4 weeks of enriched environmental training can significantly ameliorate learning and memory deficits in mice (He et al., 2017). In our previous study, it has been observed that EE training can mitigate the inflammatory responses triggered by surgical stress and enhance cognitive function postoperatively (Fan et al., 2016).

In the present study, we hypothesized that activation of TREM2 by EE could improve neurological deficits and attenuate neuroinflammation triggered by surgical trauma via the PI3K/Akt signaling pathway in a mouse model of ischemic stroke.

## Materials and methods

### Animal experiments

Eight-week-old male C57BL/6 mice weighing 20–23 g were obtained from Dashuo Experimental Animal Limited Company (Chengdu, China). As gender differences were not the primary focus of our study, female mice were not used in this experiment. The animals were housed in a temperature-controlled environment with a 12-h light/dark cycle, with free access to laboratory food and water. The animal studies were registered and approved by the Ethics Committees of Sichuan Provincial People’s Hospital Research Ethics Committee. The study was conducted in compliance with the ARRIVE guidelines. For the experimental procedures, the mice were anesthetized by inhalation of 5% isoflurane (RWD Life Technology, Shenzhen, China). Throughout the surgical interventions, anesthesia was sustained with 2% isoflurane to ensure the animals remained unconscious.

The mice were divided into the following groups: (1) Sham+SE, Sham+EE, PT + SE, and PT + EE groups; (2) SE + vehicle, SE + shRNA, EE+ vehicle, and EE + shRNA groups.

To induce ischemic stroke models, photothrombotic (PT) stroke induction was conducted as previously described (Labat-gest and Tomasi, 2013). Following anesthesia, the mice were firmly secured in a stereotaxic frame (RWD Life Science). Their body temperature was maintained at 37.0°C throughout the surgical procedure using a temperature-controlled heating pad.

**Figure 1:**
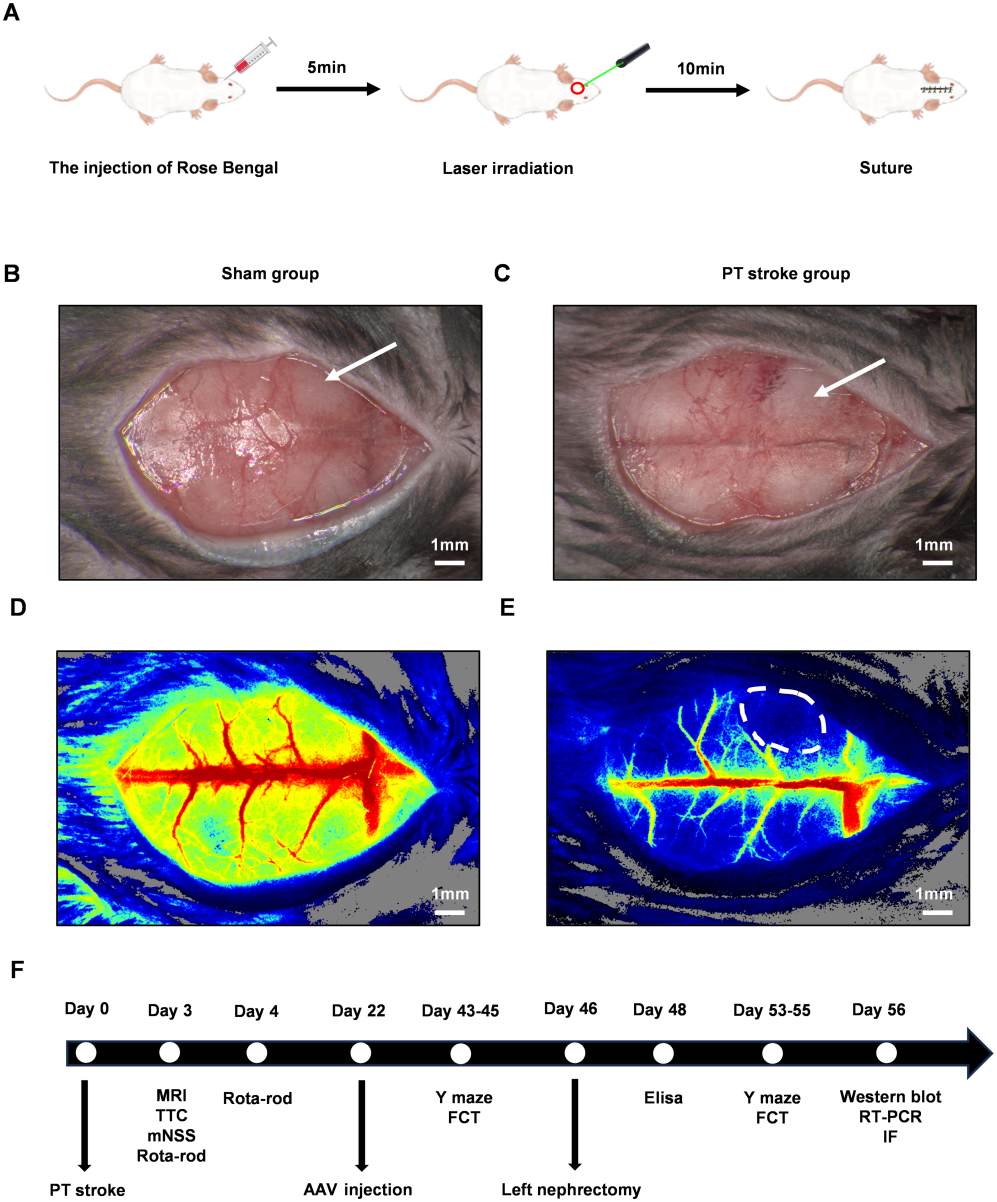
The experimental design and the verification of the cerebellar infarct region following PT stroke. The schematic diagrams of PT stroke for focal ischemic stroke model in mice were shown in **(A)**. The gross pictures after the PT stroke were shown for the sham group **(B)** and the PT group **(C)**. The white arrow indicated the target infarct site. **(D,E)** Were the pseudo-color maps of ischemic regions following PT stroke in the sham group and PT group, respectively. The white dot circled area indicated the ischemic lesion. Scale bars represented 1 mm. A Schematic of the entire experimental timeline is shown in **(F)**. PT, photothrombotic; MRI, magnetic resonance imaging; TTC, 2,3,5-Triphenyltetrazolium chloride; mNSS, modified neurological severity score; AAV, adeno-associated virus; FCT, fear conditioning test; RT-PCR, reverse transcription polymerase chain reaction; IF, immunofluorescence staining.

A 1-cm midline incision was made from the level of the eyes, and a skin retractor was used to maintain exposure of the skull. The frontal area was targeted for infarction, with the bregma serving as a reference point (coordinates: anterior/posterior: 1.5 mm, medial/lateral: 2 mm). Rose Bengal solution (Sigma-Aldrich, United States) at a dosage of 125 mg/kg was administered via slow subcutaneous injection. After a five-minute interval, the designated area was exposed to yellow-green laser light (532 nm, 40 mW/cm^2^) for a duration of 10 min, with other areas shielded from the light. The incision was then closed with sutures. Schematic diagrams are presented in Figure 1A. Immediately post-irradiation, the ischemic penumbra in the PT animals was visualized using laser speckle contrast imaging, as depicted in Figures 1B–E. The animals were closely monitored until they regained full consciousness. For the control group, sham animals underwent an identical procedure except for the omission of the subcutaneous Rose Bengal injection, thus serving as a baseline for comparison in the experiment.

To induce POCD models, all animals underwent left nephrectomy on day 46 following PT stroke induction. Briefly, mice were anesthetized and then fixed in the supine position. A midline abdominal incision was made to expose the left kidney. Blunt forceps were then used to dissect and separate the perirenal fat and connective tissue. The left kidney was subsequently extirpated after ligating the artery, vein, and ureter. The abdominal muscle layer and skin were sutured closed. Throughout the procedure, animals received continuous inhalation anesthesia, which was maintained for a total duration of 2 h to cover the entire surgical period. A schematic of the entire experimental timeline is displayed in Figure 1F.

### Stereotaxic injection of adeno-associated virus

AAV vectors encoding the mouse TREM2 gene (NCBI ID: NM_031254.4) and control AAV vectors were purchased from Hanbio (Shanghai, China). TREM2-shRNA (shRNA) or vehicle-shRNA (vehicle) with AAV vector was stereotactically injected into the bilateral hippocampi of mice with photothrombotic lesions on day 24 before left nephrectomy. Briefly, mice were anesthetized, and then these vectors were injected bilaterally into the hippocampus with the following coordinates relative to bregma: −2.3 mm anteroposterior, ±1.8 mm mediolateral, and − 2 mm dorsoventral. Each site was injected with 1 μL (4 × 10^9^ vg/site) of diluted AAV vectors over a 10-min period. The efficacy of AAV-mediated TREM2 silencing was verified on day 21 after injection.

### Enriched environment housing

Following the induction of PT stroke, mice were randomly assigned to either EE or SE housing without stratification. The EE was composed of larger cages measuring 43.1 cm × 22.8 cm × 19.5 cm, equipped with a variety of stimulating objects such as climbing ladders, running wheels, mazes, seesaws, and balls. The toys and bedding were refreshed every 3 days to maintain a dynamic and engaging environment. In contrast, smaller cages measuring 27.9 cm × 15.2 cm × 11.4 cm, devoid of any additional toys, were set as SE. All animals had free access to water and food.

### Measurement of infarct volume

The formation of the infarct focus was preliminarily evaluated using magnetic resonance imaging (MRI, Bruker BioSpec 70/30USR, Germany). For a quantitative calculation of the infarct volume, the brain sections were incubated in 2% 2,3,5-Triphenyltetrazolium chloride (TTC, Sigma-Aldrich) for 20 min at 37°C. The infarcted and non-infarcted areas were then analyzed using Image J software (National Institutes of Health, United States). The percentage of infarct volume was calculated as the total volume of white infraction / total volume of brain section × 100%.

### Rota-rod test

The mice were tested using the rota-rod test to assess their motor coordination abilities. The animals were placed on a rotating rod that accelerated smoothly from 4 to 40 rpm per min over a 5-min period. The length of time taken for the mice to fall off the rod was recorded, with a maximum cutoff time of 300 s. Prior to the establishment of the ischemic stroke model, the initial drop latency time was documented as the baseline measurement.

### Modified neurological severity score

The mNSS test, which consisted of five tasks including motor, sensory, balance, and reflex functions, was performed to assess their neurological functions. The scores were graded from 0 to 18 (0 = normal function; 1–7 = mild deficit; 8–12 = moderate deficit; 13–18 = severe deficit), and higher scores reflected greater neurological injury.

### Y-maze test

Short-term cognitive memory and exploratory behavior were measured using a Y-maze apparatus (25 cm long × 8 cm wide × 15 cm high, oriented at 120°, BrainScience Idea, Osaka, Japan). Mice were placed at the center of the Y-maze and allowed to freely explore the three arms for a duration of 5 min. The spontaneous alternation behavior (SAB) was calculated as the percentage of spontaneous alternation: (the number of spontaneous alternations) / (total number of arms crossed – 2) × 100%.

In the Y-maze novel arm test, the three arms were randomly assigned to be the novel arm, the start arm, and the other arm. During the free-exploration phase, mice were allowed to explore the start arm and the other arm for a 10-min session while the novel arm was blocked. After this, the mice were returned to their cages for a 1-h interval. In the test phase, the mice were placed back in the start arm with access to the novel arm for a 5-min session. The time spent in each arm was recorded, and the novel arm entries were calculated as follows: time spent in the novel arm / total time× 100%.

### Fear conditioning test

The fear conditioning test, comprising both contextual and tone tests, was designed to assess hippocampal-dependent and non-hippocampal-dependent cognitive functions, respectively. Before conditioning, the testing chamber (Ugo Basilie, Italy) was sanitized with 75% ethyl alcohol. The mice were gently put into the chamber to adapt to the surroundings for 2 min. Subsequently, they were subjected to two pairings of tone and foot shock (tone at 2,000 Hz, 85 dB, for 30 s; foot shock at 0.8 mA, for 2 s) with a 60-s intertrial interval. After a 24-h interval, mice were randomly assigned to either the contextual test or the tone test, and their freezing behavior was recorded. In the contextual test, mice were returned to the same test chamber without any shock for a duration of 8 min. For the tone test, mice were placed in a differently lit chamber, which had been wiped with white vinegar to provide a distinct olfactory context. Following a 3-min adaptation period to the test box, the sound stimulation (2,000 Hz, 85 dB, for 30 s) was administered for 4.5 min. The entire test, including the adaptation period, lasted 8 min.

### Enzyme-linked immunosorbent assay

The levels of inflammation-associated cytokines in the hippocampi, including IL-1β, IL-6, TNF-α, IL-4, and IL-10, were quantified using specific ELISA kits (R&D systems, United States). All experimental procedures were performed following the manufacturer’s instructions.

### Immunofluorescence staining

The hippocampi were fixed in 4% paraformaldehyde and embedded in paraffin for immunofluorescence staining. After antigen retrieval with EDTA, the paraffin sections were washed with phosphate-buffered solution (PBS) and then blocked with 5% bovine serum albumin at room temperature for 1 h. Following this, the sections were incubated with a mouse anti-TREM2 primary antibody (1:1,000, 68723-1-Ig, Proteintech) overnight at 4°C. The next day, sections were incubated with a goat anti-mouse secondary antibody (1:500, RGAM004, Proteintech) for 1 h at room temperature after being washed with PBS. For immunofluorescence double staining, these sections were then incubated with the following primary antibodies overnight at 4°C: rabbit anti-Iba-1 (1:400, 26177-1-AP, Proteintech), rabbit anti-GFAP (1:2500, 16825-1-AP, Proteintech), and rabbit anti-MAP2 (1:2,500, 17490-1-AP, Proteintech) overnight at 4°C, respectively. After being washed with PBS, sections were incubated with a goat anti-rabbit secondary antibody (1:500, RGAR002, Proteintech) for 1 h at room temperature. Finally, the sections were stained with 4′,6-diamidino-2-phenylindole (DAPI) to locate the nucleus for 5 min and then immediately sealed with an anti-fluorescence quenching agent. Stained images were obtained through the LSM700 confocal microscopy system (ZEISS, Germany). The co-localization of TREM2 with Iba-1, GFAP, and MAP2 was quantified using the Pearson correlation coefficient (PCC). The PCC measures the linear correlation between the fluorescence signals of the two markers, where a PCC of 1 indicates a perfect positive correlation, 0 indicates no correlation, and − 1 indicates a perfect negative correlation. To determine if the PCC was significantly greater than 0 (which would indicate no correlation), a one-sample, one-tailed student’s *t*-test was employed (Paul et al., 2021).

### Western blotting analysis

The total proteins of the hippocampi were extracted by using a tissue protein extraction kit (BC3711, Solarbio, Beijing, China) according to the manufacturer’s instructions. Equal amounts of proteins were separated by sodium dodecyl sulfate-polyacrylamide gel electrophoresis (SDS-PAGE) followed by transfer to polyvinylidene fluoride (PVDF) membranes. After being blocked with 5% milk for 60 min at room temperature, PVDF membranes were incubated at 4°C overnight with the following primary antibodies: rabbit anti-TREM2 antibody (1:1,000, 27599-1-AP, Proteintech), mouse anti-PI3K antibody (1:5,000, 60225-1-Ig, Proteintech), rabbit anti-phosphor-PI3K antibody (1:1000, HA721672, Huabio), rabbit anti-AKT antibody (1:2,000, 10176-2-AP, Proteintech), rabbit anti-phospho-AKT antibody (1:5,000, 80455-1-RR, Proteintech), rabbit anti-DAP12 antibody (1:1,000, EPR24244-76, Abcam), rabbit anti-BDNF antibody (1:600 dilution, 25699-1-AP, Proteintech), and mouse anti-*β*-actin antibody (1:20,000 dilution, 66009-1-Ig, Proteintech). PVDF membranes were washed with TBST buffer three times and then incubated with secondary antibodies for 1 h at room temperature on the next day. The bands on PVDF membranes were visualized in a chemiluminescence machine (Bio-Rad, United States) using an enhanced chemiluminescence kit (Solarbio, China). The relative density of the protein images was analyzed using ImageJ software.

### Reverse transcription polymerase chain reaction

The total RNA of hippocampi was collected with Trizol reagent (Sigma-Aldrich) according to the operation instructions. The reverse transcription of the total RNA was completed by using the HiScript II Q RT SuperMix for the qPCR kit (Vazyme Biotech, China). The synthesized cDNA templates were used to do quantitative PCR by using SYBR Green PCR reagents (Bio-Rad). The ΔΔCt (the threshold cycle) values were calculated, and the results were expressed as the ratio of the mRNA copies of the TREM2, DAP12, and BDNF genes to that of the *β*-actin genes (reference gene). All data was presented in the fold change compared to the control group.

The primers involved in our study were shown as follows: 5′-ACTGGCTTGGTCATCTCTTTTCT-3′ (forward) and 5′-GTGTTGAGGGCTTGGGACA-3′ (reverse) for TREM2; 5′-CCTCCTGGTGCCTTCTGTTC-3′ (forward) and 5′-AGTCGCATCTTGGGAAAGTGT-3′ (reverse) for DAP12; 5′-TATTAGCGAGTGGGTCACAGC-3′ (forward) and 5′-ATTGCGAGTTCCAGTGCCTT-3′ (reverse) for BDNF; 5′-GGCTGTATTCCCCTCCATCG-3′ (forward) and 5′-CCAGTTGGTAACAATGCCATGT-3′ (reverse) for β-actin.

### Statistical analyses

The sample size was based on previous pilot studies and literature. All data were shown as the mean and standard deviation (mean ± SD). Statistical evaluation of the data was performed by one-way analysis of variance (ANOVA), followed by Tukey multiple-comparison *post hoc* analysis. Statistical significance was defined as *p* < 0.05. All statistical graphs were performed with Graph Pad Prism (Graph Pad Software 6.0, San Diego, CA, United States).

## Results

### EE protected against neurological dysfunctions induced by ischemic stroke in mice

**Figure 2:**
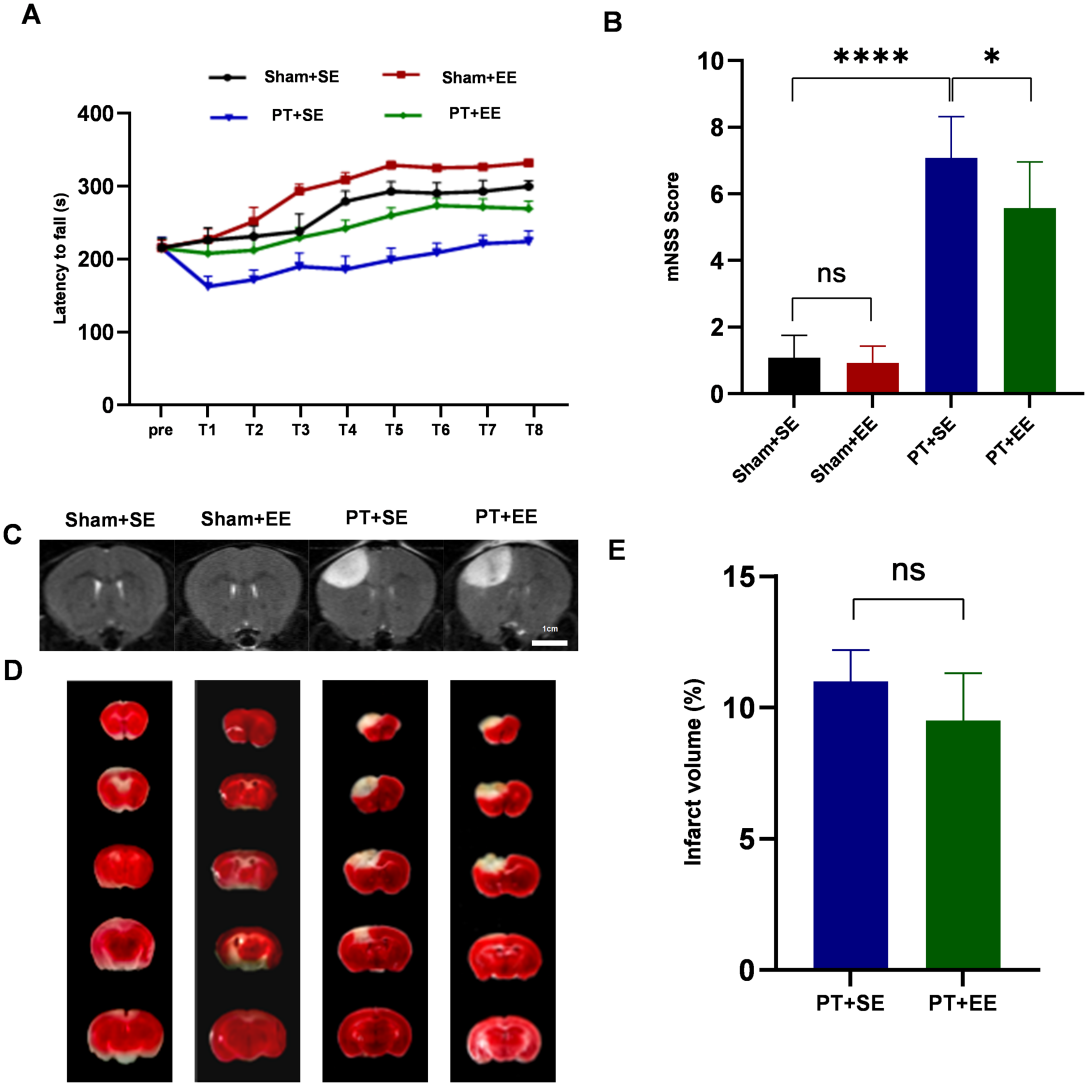
EE protects against brain damage after an ischemic stroke. **(A)** The time of latency to fall in the rota-rod test during the 2-day training (*n* = 18). **(B)** The brain damage was evaluated by mNSS scores (*n* = 12). **(C)** The T2-weighted images of whole-brain MRI on day 3 after the PT stroke showed the infarct area (increased signal intensity). **(D)** 2,3,5-Triphenyltetrazolium chloride (TTC) staining of brain slices 3 days after PT stroke showed the infarct area (white). **(E)** The relative proportion and quantification of infarct volume by TTC staining (*n* = 3). ns: no significant, *p < 0.05, ***p < 0.001, ****p < 0.0001. Error bars were represented as mean ± SD.

To determine the neuroprotective effects of EE *in vivo*, the rota-rod test and mNSS were applied to evaluate the neurological functions of mice after PT stroke. On days 3 and 4 after ischemic stroke, the latency to fall was calculated 8 times from 4 consecutive measurements with an interval of 1 h a day. As presented in Figure 2A, mice in all PT groups maintained their balance on the rota-rod apparatus for a shorter time than the sham groups. Mice in the PT + EE group had longer latency times compared with the PT + SE group. Similarly, mice in the Sham+EE group had longer latency times than mice in the Sham +SE group. In the mNSS test, mice in the PT + SE group showed higher scores when compared with the PT + EE group (*p* < 0.05) and the Sham+SE group (*p* < 0.0001) on day 3 after PT stroke (Figure 2B).

Meanwhile, we performed a whole-brain MRI to detect the ischemic foci on day 3 after the ischemic stroke. The T2-weighted images characterized by increased signal intensity compared to the surrounding brain tissue could be observed in the PT + SE group and PT + EE group (Figure 2C). In TTC staining, a white area was observed in the PT + SE group and PT + EE group, which represented cerebral infarction (Figure 2D). However, infarct volume had no significant difference between the PT + SE group and PT + EE group according to the TTC staining (*p* > 0.05, Figure 2E). The results indicated that EE ameliorated neurological dysfunctions induced by ischemic brain injury and promoted motor coordination ability in mice without brain damage.

### EE improved neurological impairment exacerbated by ischemic stroke after surgical trauma

**Figure 3:**
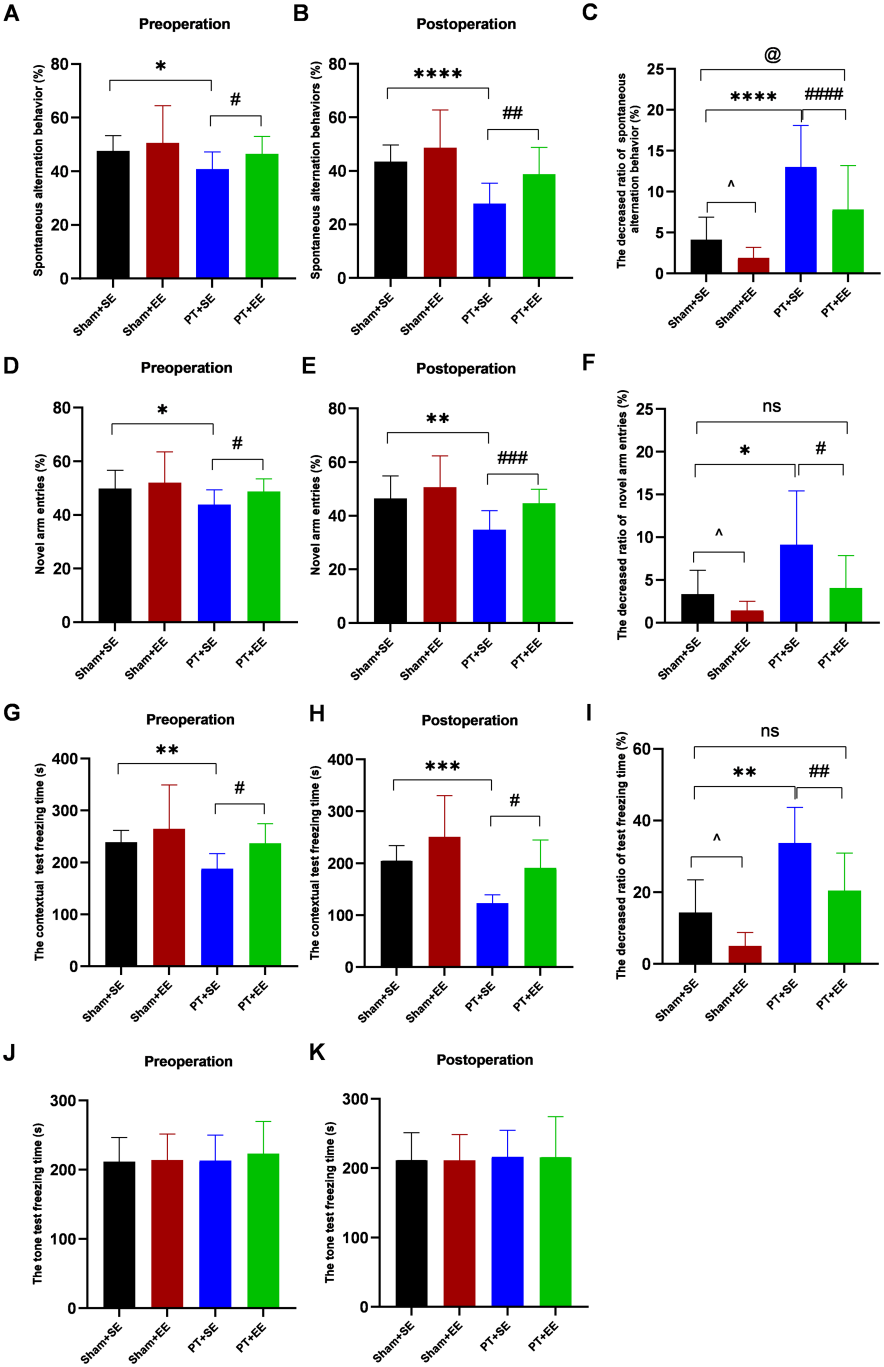
The results of behavioral tests before and after surgery in mice with ischemic stroke. **(A–C)** The percentage of spontaneous alteration in the Y maze test (*n* = 12). **(D–F)** The percentage of novel arm entries in the Y maze test (*n* = 12). **(G–I)** The freezing time in contextual test (*n* = 6). **(J–K)** The freezing time in tone test (*n* = 6). ns: no significant; *p < 0.05, **p < 0.01, ***p < 0.001, ****p < 0.0001, the Sham+SE group vs. the PT + SE group; #*p* < 0.05, ##*p* < 0.01, ###*p* < 0.001, the PT + SE group vs. the PT + EE group; ^*p* < 0.05, the Sham+SE group vs. the Sham+EE group; @*p* < 0.05, Sham+SE group vs. the PT + EE group. Error bars were represented as mean ± SD.

To investigate the effect of EE on surgery-induced cognitive dysfunctions in mice with ischemic stroke, the Y-maze test was used to assess the memory functions and spatial learning abilities of mice on days 7–10 after left nephrectomy. As shown in Figure 3A, the SAB ratio showed a significant decrease in the PT + SE group when compared to the Sham+SE (*p* < 0.05) group and PT + EE group (*p* < 0.05) before surgery. And surgical trauma enlarged their difference (*p* < 0.0001 for Sham+SE vs. PT + SE, *p* < 0.01 for PT + SE vs. PT + EE, Figure 3B). The decreased SAB ratio was higher in the PT + SE when compared to the Sham+SE group (*p* < 0.0001) and PT + EE (*p* < 0.0001, Figure 3C). These data indicated that surgical trauma exacerbated the existing memory impairment in mice with ischemic stroke, and EE improved their memory functions. Meanwhile, the decreased SAB ratio was lower in the Sham+EE when compared to the Sham+SE group (*p* < 0.05), which indicated that EE was also helpful for improving memory in mice without brain damage. Similar to the memory functions, the Y-maze novel arm test showed the positive effects of EE on the spatial learning abilities of mice with ischemic stroke. As shown in Figure 3D, the novel arm entries showed a significant decrease in the PT + SE group when compared to the Sham+SE (*p* < 0.05) group and PT + EE group (*p* < 0.05) before surgery. And a larger decrease could be observed after surgery (*p* < 0.01 for Sham+SE vs. PT + SE, *p* < 0.001 for PT + SE vs. PT + EE, Figure 3E). The decreased novel arm entries were higher in the PT + SE when compared to the Sham+SE group (*p* < 0.05) and PT + EE (*p* < 0.05, Figure 3F). The above findings indicated that surgical trauma exacerbated cognitive dysfunctions induced by ischemic stroke, while EE played a role in attenuating learning and memory impairment.

To explore whether the hippocampus was involved in regulating cognitive functions, the mice were tested by the contextual test and tone test on days 7–10 after left nephrectomy. In the contextual test, the freezing time was shorter in the PT + SE group when compared with the Sham+SE group (*p* < 0.01 for preoperation, *p* < 0.001 for postoperation) and PT + EE (*p* < 0.05 for preoperation, p < 0.05 for postoperation, Figures 3G,H). Figure 3I showed that the PT + SE group exhibited the greatest change ratio in freezing time among all groups. However, there was no significant difference among all groups in the tone-related fear test (Figures 3J–K). It indicated that both the ischemic stroke-induced impairment and surgery-induced impairment were hippocampus-dependent.

### EE suppressed neuroinflammation in the hippocampus after surgery

**Figure 4:**
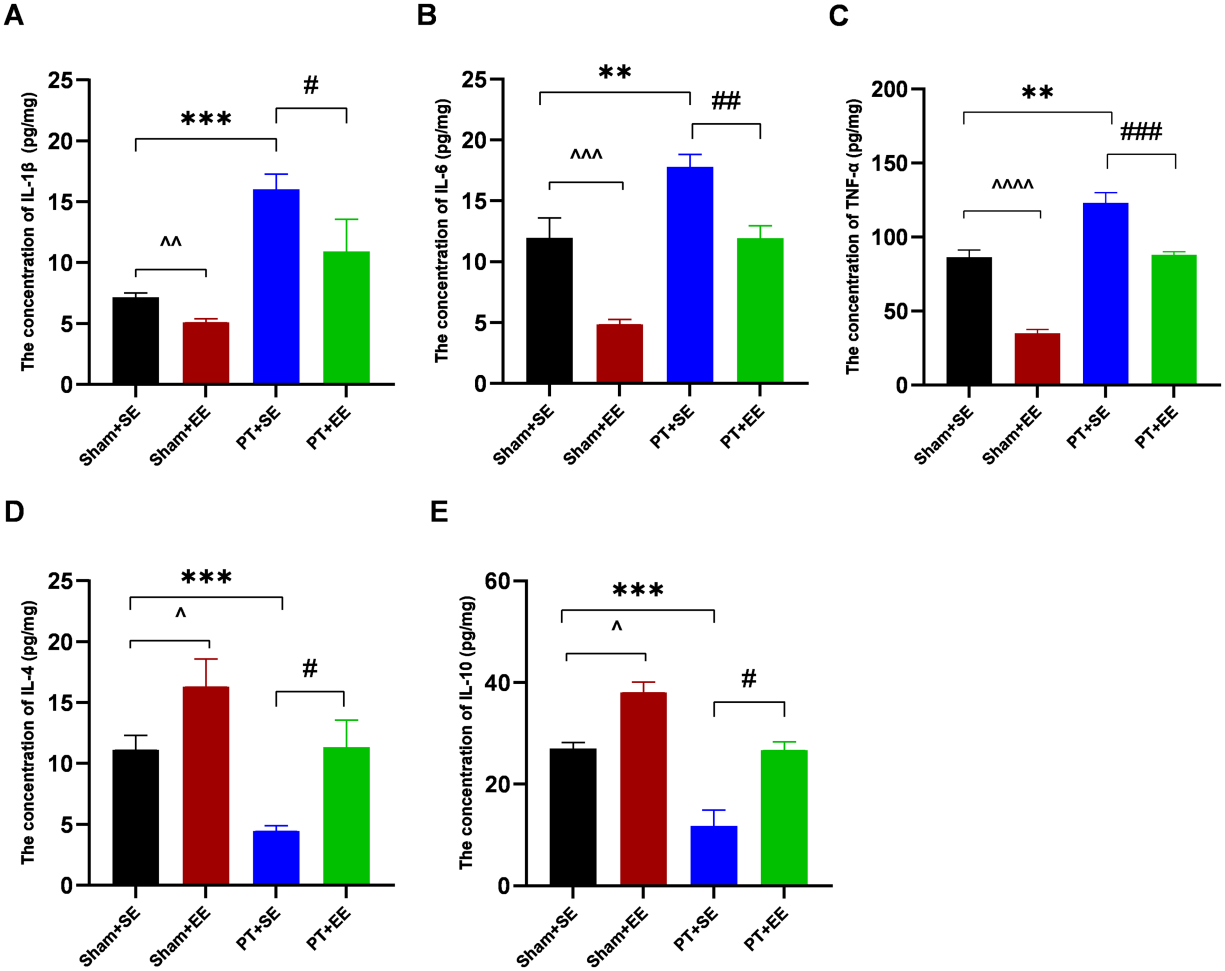
The inflammation level in the hippocampus after surgery in mice with ischemic stroke. **(A–E)** The concentrations of inflammation-associated factors in the hippocampus in mice with ischemic stroke at 24 h after surgery (*n* = 3). **p < 0.01, ***p < 0.001, the Sham+SE group vs. the PT + SE group; #*p* < 0.05, ##*p* < 0.01, ###*p* < 0.001, the PT + SE group vs. the PT + EE group; ^*p* < 0.05, ^^*p* < 0.01, ^^^*p* < 0.001, ^^^^*p* < 0.0001, the Sham+SE group vs. the Sham+EE group. Error bars were represented as mean ± SD.

Twenty-four hours after surgery, the expression level of inflammation-associated factors in the hippocampus was significantly higher in the PT + SE group when compared to the Sham+SE group and PT + EE group (IL-1β: *p* < 0.001 for sham+SE *vs*. PT + SE, *p* < 0.05 for PT + SE *vs*. PT + EE; IL-6: *p* < 0.01 for sham+SE *vs*. PT + SE, p < 0.01 for PT + SE *vs*. PT + EE; TNF-*α*: *p* < 0.01 for sham+SE *vs*. PT + SE, *p* < 0.001 for PT + SE *vs*. PT + EE; Figures 4A–C). And compared with the Sham+SE group, the concentrations of IL-6 (*p* < 0.001) and TNF-α (*p* < 0.0001) were significantly decreased in the Sham+EE group (Figures 4A–C). However, opposite changing trends were observed in the expressions of proinflammatory factors, including IL-4 and IL-10 (Figures 4D,E). These results indicated that the development of POCD had a strong correlation with high-grade inflammation in the hippocampus, and EE inhibited the inflammatory reaction to improve neurological dysfunctions induced by cerebral injury and surgery.

### EE upregulated the expression of TREM2 after surgical trauma

**Figure 5:**
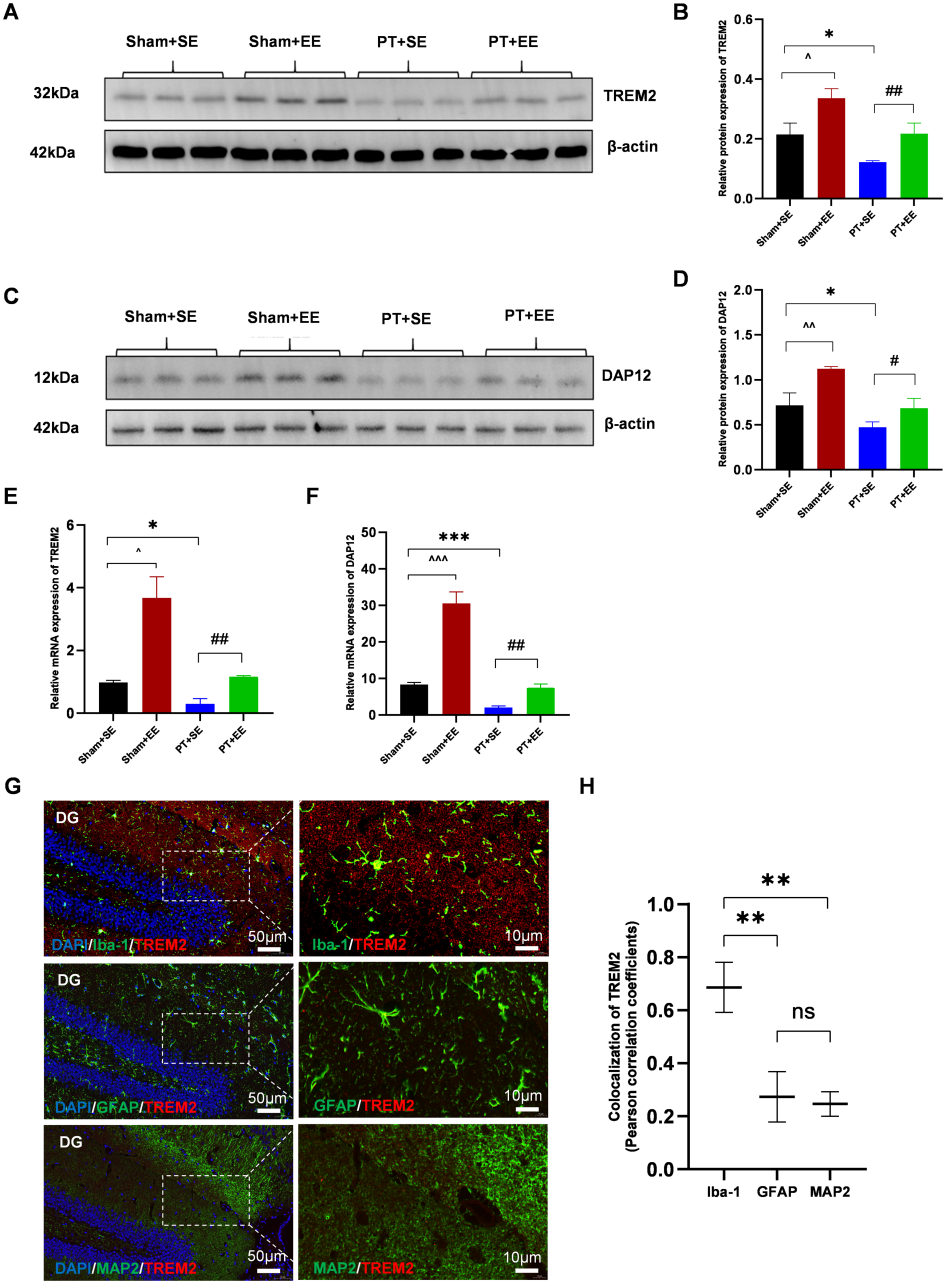
EE upregulated the expression of TREM2 in the hippocampus after surgery in mice with ischemic stroke. **(A,B)** The expression of TREM2 protein in the hippocampus on day 10 after surgery (*n* = 3). **(C,D)** The expression of DAP12 protein in the hippocampus on day 10 after surgery (*n* = 3). **(E)** The expression of TREM2 mRNA in the hippocampus on day 10 after surgery (*n* = 3). **(F)** The expression of DAP12 mRNA in the hippocampus on day 10 after surgery (*n* = 3). **(G)** The immunofluorescence of the cellular localization of TREM2 in the hippocampus of mice in the Sham+EE group on day 10 after surgery (*n* = 3). DG: dentate gyrus. Scale bars represented 10 μm and 50 μm. ns: no significant; *p < 0.05, ***p < 0.001, the Sham+SE group vs. the PT + SE group; #*p* < 0.05, ##*p* < 0.01, the PT + SE group vs. the PT + EE group; ^*p* < 0.05, ^^^*p* < 0.001, the Sham+SE group vs. the Sham+EE group. Error bars were represented as mean ± SD.

To explore the effect of EE on the endogenous expression level of TREM2 in the hippocampus, we performed RT-PCR and the western blot analysis on day 10 after surgery. The results of the western blot showed that ischemic stroke downregulated the expression of TREM2 at the protein level in the hippocampus while EE reversed its effect (*p* < 0.05 for Sham+SE *vs*. PT + SE; *p* < 0.05 for Sham+SE *vs*. Sham+EE; *p* < 0.01 for PT + SE *vs*. PT + EE; Figures 5A,B). DAP12, as the obligate adaptor of TREM2, its expression level at the protein level showed a similar trend coinciding with that of TREM2 (Figures 5C,D). Meanwhile, the results of RT-PCR further confirmed the changing trend of TREM2 and DAP12 at the mRNA level (Figures 5E,F).

It is reported that TREM2 is primarily expressed by microglia in the CNS, with expression levels significantly higher than in neurons or other cells (Deczkowska et al., 2020). Therefore, double immunofluorescence staining was performed to determine the cellular localization of TREM2 in the hippocampus of mice in the Sham+EE group on day 10 after surgery.

The results showed that the fluorescence signal of TREM2 was highly colocalized with that of Iba-1 (microglia), whereas no significant overlapping fluorescence signals were observed between TREM2 and GFAP (astrocytes) or between TREM2 and MAP2 (neurons) in the dentate gyrus (DG) (Figures 5G,H). These results indicated that the upregulation of TREM2 on microglia in the hippocampus played an important role in neuroprotection against surgical trauma.

### EE activated the PI3K/Akt signaling pathway

**Figure 6:**
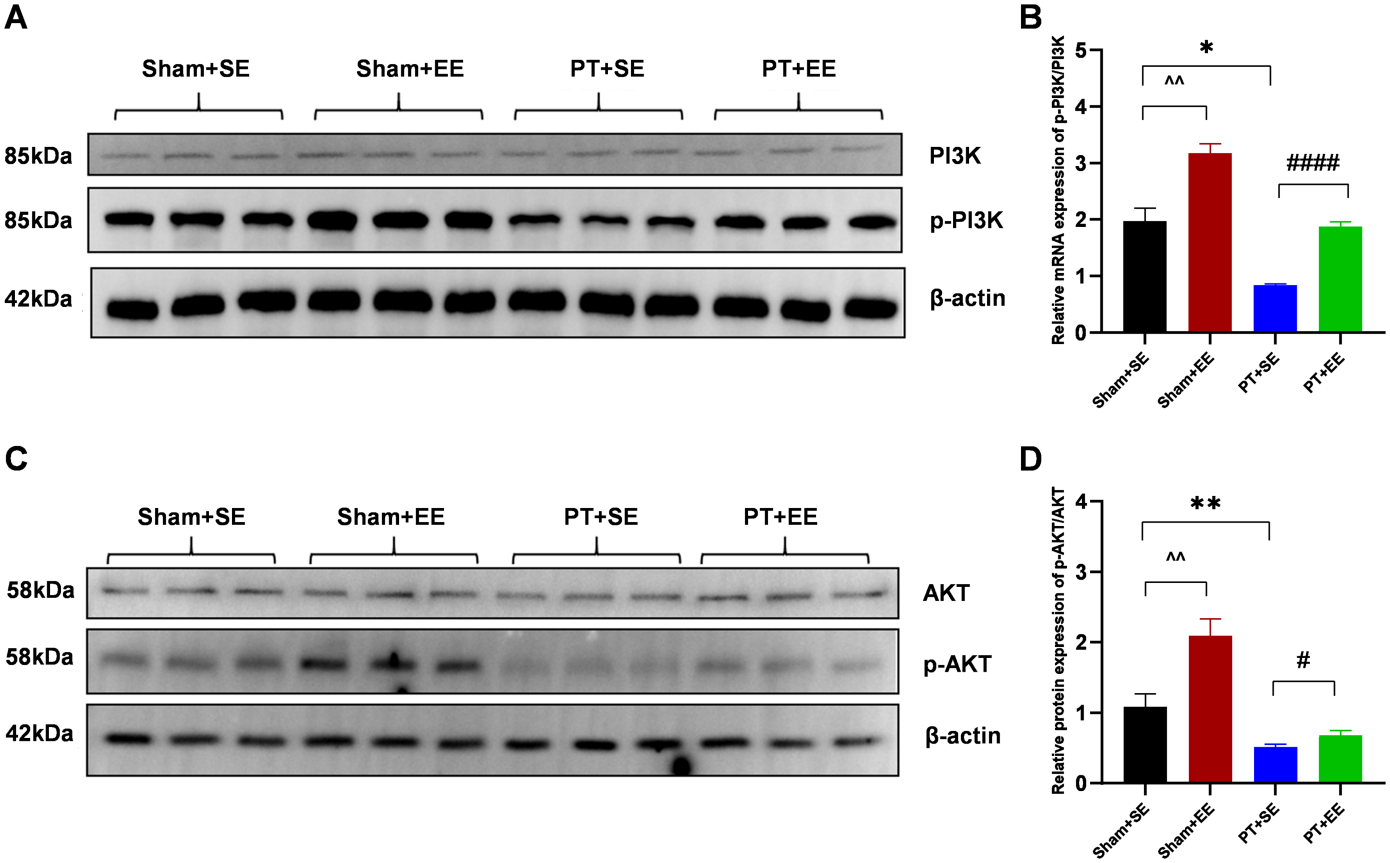
EE activated the PI3K/Akt signaling pathway in the hippocampus after surgery in mice with ischemic stroke. **(A)** The results of the western blot of p-PI3K and PI3K. **(B)** The protein p-PI3K / PI3K ratio in the hippocampus on day 10 after surgery (*n* = 3). **(C)** Western bolt results of p-AKT and AKT. **(D)** The protein p-AKT/ AKT ratio in the hippocampus on day 10 after surgery (*n* = 3). *p < 0.05, **p < 0.01, the Sham+SE group vs. the PT + SE group; #*p* < 0.05, ####*p* < 0.0001, the PT + SE group vs. the PT + EE group; ^^*p* < 0.01, the Sham+SE group vs. the Sham+EE group. Error bars were represented as mean ± SD.

To explore the possible mechanism of how EE improved POCD in mice with ischemic stroke, we focused on the PI3K/Akt signaling pathway, which is important in regulating the inflammatory reaction. We observed that after surgery, the p-PI3K / PI3K ratio in the EE groups was significantly higher than that in the SE groups (*p* < 0.01 for sham+SE *vs*. sham+EE; *p* < 0.0001 for PT + SE *vs*. PT + EE; Figures 6A,B). Additionally, the downregulation of the p-PI3K / PI3K ratio was found in the hippocampus of mice with ischemic stroke (*p* < 0.05 for sham+SE *vs*. PT + SE, Figures 6A,B). The same trend was found in the protein p-Akt /Akt ratio, as shown in Figures 6C,D. The above data demonstrated that EE reversed the development of POCD via activating the PI3K/Akt signaling pathway, and ischemic stroke aggravated neurologic impairment by inhibiting the phosphorylation of key proteins.

### The knockdown of TREM2 abolished the neuroprotective effects of EE in mice with ischemic stroke

In this study, AAV-TREM2-shRNA was injected in C57BL/6 mice to knock down the expression of TREM2 in the hippocampal tissue. To determine the knockdown efficiency of AAV-TREM2-shRNA in each group, the expression of TREM2 in the hippocampal tissue was detected by western blot analysis and RT-PCR on day 21 after the stereotaxic injection of AAV-TREM2-shRNA.

**Figure 7:**
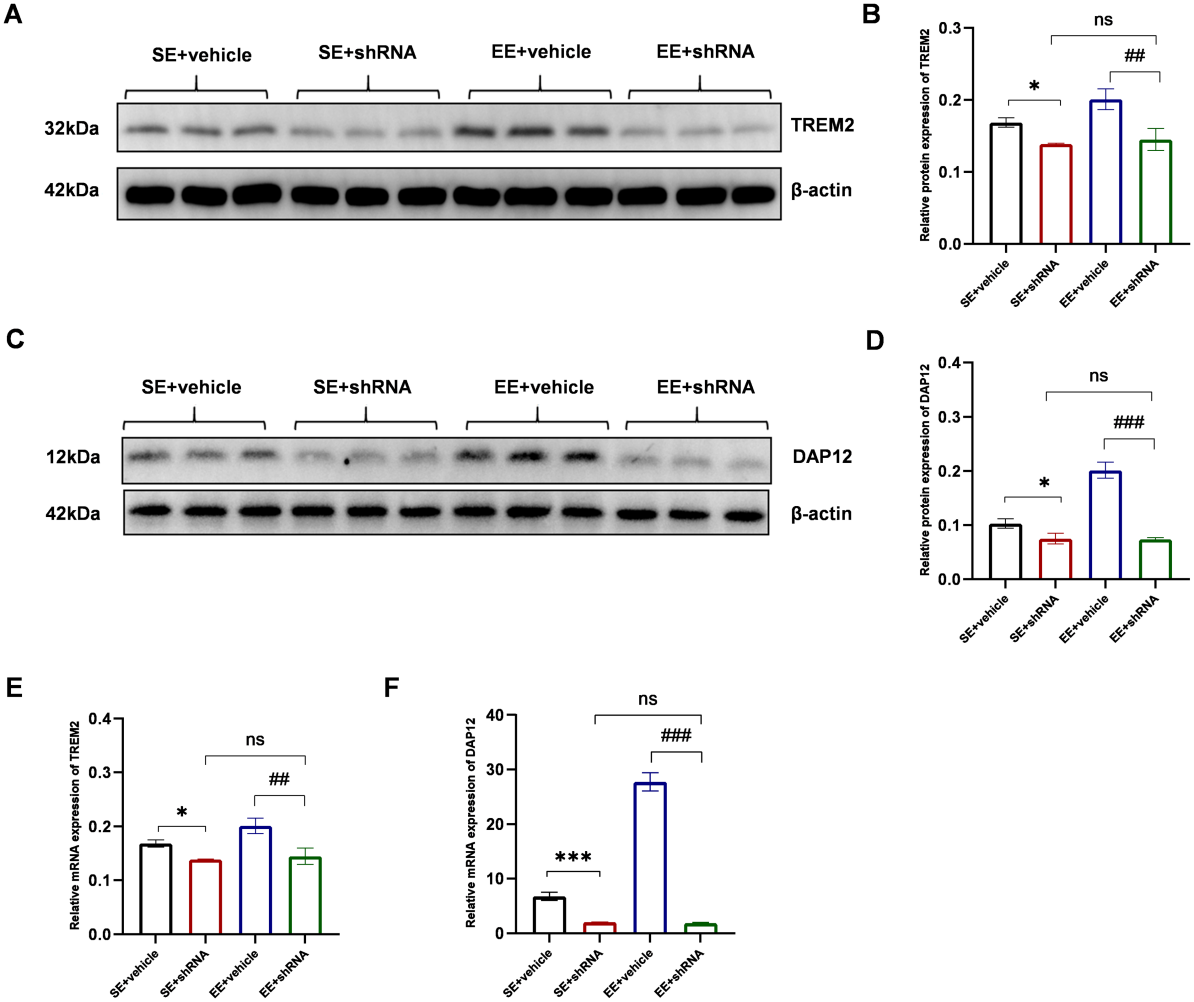
TREM2 shRNA successfully decreased the expression of TREM2 in the hippocampus on day 21 after the injection of TREM2 shRNA. **(A,B)** The expression of TREM2 protein in the hippocampus (*n* = 3). **(C,D)** The expression of DAP12 protein in the hippocampus (*n* = 3). **(E)** The expression of TREM2 mRNA in the hippocampus (*n* = 3). **(F)** The expression of DAP12 mRNA in the hippocampus. ns: no significant; *p < 0.05, ***p < 0.001, the SE + vehicle group vs. the SE + shRNA group; ##*p* < 0.01, ###*p* < 0.001, the EE+ vehicle group vs. the EE+ shRNA group. Error bars were represented as mean ± SD.

The results of the western blot showed that the expression of TREM2 at the protein level showed a significant decrease in the hippocampus after the administration of TREM2-shRNA (*p* < 0.05 for SE+ vehicle *vs*. SE+ shRNA and *p* < 0.01 for EE+ vehicle *vs*. EE+ shRNA, Figures 7A,B). Moreover, the expression level of the TREM2 protein showed no significant difference between the SE+ shRNA group and the EE+ shRNA group (*p* > 0.05, Figures 7A,B). The same trend was found in the expression level of the DAP12 protein, as Figures 7C,D shows. The results of RT-PCR further confirmed the knockdown of TREM2 at the mRNA level (Figures 7E,F).

**Figure 8:**
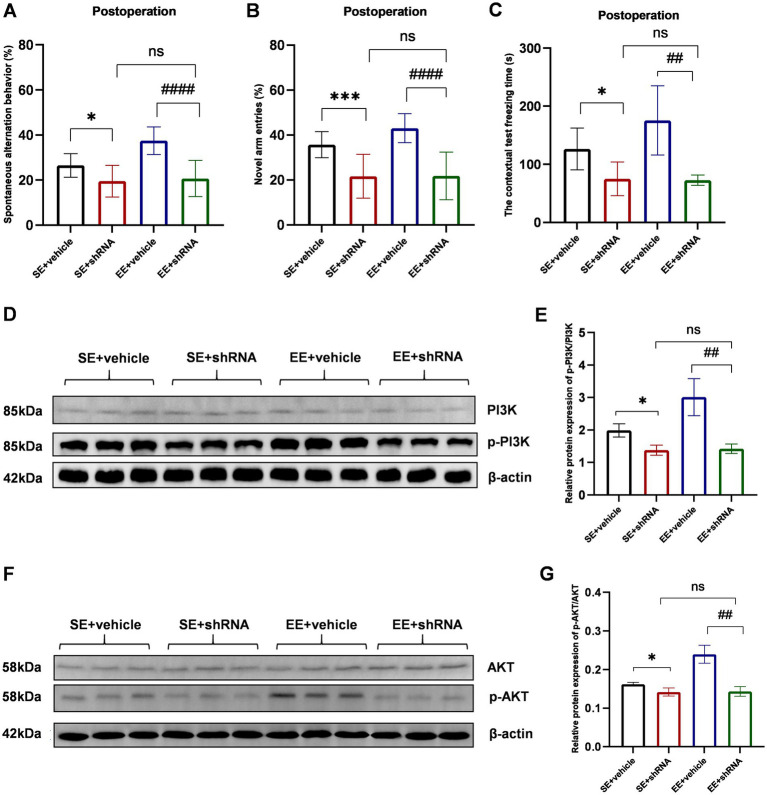
The knockdown of TREM2 abolished the neuroprotective effects of EE and inhibited the PI3K/Akt signaling pathway in the hippocampus of mice with ischemic stroke after surgery. **(A–C)** The behavioral tests of each group (*n* = 6). **(D)** Western bolt results of p-PI3K and PI3K. **(E)** The protein p-PI3K / PI3K ratio in the hippocampus on day 10 after surgery (*n* = 3). **(F)** Western bolt results of p-AKT and AKT. **(G)** The protein p-AKT/AKT ratio in the hippocampus on day 10 after surgery (*n* = 3). ns: no significant; *p < 0.05, ***p < 0.001, the SE + vehicle group vs. the SE + shRNA group; ##*p* < 0.01, ####*p* < 0.0001, the EE+ vehicle group vs. the EE+ shRNA group. Error bars were represented as mean ± SD.

The knockdown of TREM2 in the hippocampus induced severe neurological deficits assessed by the Y-maze spontaneous alternation test (*p* < 0.05 for SE+ vehicle *vs*. SE+ shRNA, *p* < 0.0001 for EE+ vehicle *vs*. EE+ shRNA, Figure 8A), Y-maze novel arm test (*p* < 0.001 for SE+ vehicle *vs*. SE+ shRNA, p < 0.0001 for EE+ vehicle *vs*. EE+ shRNA, Figure 8B), and contextual test (*p* < 0.05 for SE+ vehicle *vs*. SE+ shRNA, p < 0.01 for EE+ vehicle *vs*. EE+ shRNA, Figure 8C). Meanwhile, mice in the SE+ shRNA group had similar performance in behavioral tests compared with mice in the EE + shRNA group (*p* > 0.05, Figures 8A–C). These data indicated that the TREM2-knockdown in the hippocampus abolished the neuroprotective effects of EE after surgery.

The TREM2-knockdown with TREM2-shRNA significantly decreased the phosphorylation level of PI3K and Akt in shRNA groups compared with vehicle groups (p-PI3K/PI3K: *p* < 0.05 for SE+ vehicle *vs*. SE+ shRNA, *p* < 0.01 for EE+ vehicle *vs*. EE+ shRNA; p-AKT/AKT: *p* < 0.05 for SE+ vehicle *vs*. SE+ shRNA, *p* < 0.01 for EE+ vehicle *vs*. EE+ shRNA, Figures 8D–G). Meanwhile, the expression level of p-PI3K and p-AKT showed no difference between the SE+ shRNA group and the EE+ shRNA group (*p* > 0.05, Figures 8D–G). Similar expression levels of IL-1β, IL-6, TNF-α, IL-4, and IL-10 were observed in the two groups (*p* > 0.05, Supplementary Figures S1A–E). According to the above data, it could be speculated that the TREM2-knockdown inhibited the activation of the PI3K/Akt signaling pathway to abolish the neuroinflammation inhibitory effects of EE in the development of POCD.

## Discussion

Neuroinflammation plays a significant role in neurodegenerative diseases and has been established as an independent risk factor for the development of POCD (Li et al., 2022; Qin et al., 2022; Yang et al., 2022). In our experiment, the post-surgical assessment of proinflammatory cytokines, including IL-1β, IL-6, and TNF-α in the hippocampal tissue, revealed that the inflammatory response in the CNS of mice with stroke was markedly higher than that in mice undergoing sham operations. During cerebral ischemia, alterations in endothelial shear stress and hemorheological changes due to vascular occlusion rapidly initiate an inflammatory cascade (Jurcau and Simion, 2021). The sustained inflammatory response following an infarct may disrupt the blood–brain barrier’s integrity, permitting peripheral inflammatory cells and factors activated by surgical stress to infiltrate the CNS and exacerbate the inflammatory response (Liu et al., 2023). Based on this perspective, interventions that target neuroinflammatory responses, including the direct administration of anti-inflammatory medications, the blockade of inflammatory signaling pathways, and the modulation of microglial activity, have attracted increased attention in the prevention and treatment of POCD (Huang et al., 2020; Li et al., 2020; Cho et al., 2021).

TREM2 is pivotal in modulating immune responses, inflammation, and neuroprotection, positioning it as a promising therapeutic target for neurodegenerative diseases such as Alzheimer’s, Parkinson’s, and multiple sclerosis (Cignarella et al., 2020; Zhang et al., 2023; Huang et al., 2024). The activation of TREM2 can mitigate inflammatory responses and enhance microglial phagocytosis, thereby ameliorating neurological function and reducing neuroinflammation and neuronal apoptosis (Liu et al., 2022). Our research corroborates these effects, as mice in EE demonstrated superior behavioral performance post-stroke and post-surgical stimulation compared to those in SE, with concurrent elevated TREM2 expression in their hippocampal tissue. The activated PI3K/Akt signaling pathway induced by TREM2 mediates the shift of microglia from the M1 to the M2 phenotype in response to CNS inflammation, thereby improving cognitive impairment (Wang et al., 2020). Our study further identified the activation of the PI3K/Akt signaling pathway, with the expression levels of phosphorylated proteins being positively correlated with TREM2 expression levels. Additionally, the knockdown of endogenous TREM2 by TREM2 shRNA exacerbated neurological deficits and reduced TREM2 expression in ischemic stroke mice. Moreover, TREM2 knockdown reversed the beneficial effects of EE on the expression of proinflammatory factors and key proteins within the PI3K/Akt signaling pathway. Ultimately, our findings suggest that TREM2 activation induced by EE mitigated neuroinflammation and POCD in ischemic stroke mice, an effect that was, at least in part, mediated by the activation of the PI3K/Akt signaling pathway.

Microglia are essential in hippocampal neuroinflammation following aseptic trauma, where they influence cognitive function by modulating neural activity and synaptic plasticity through the release of various cytokines (Feng et al., 2017; Lecca et al., 2022). It was reported that TREM2 was expressed not only on microglia but also on astrocytes and neurons in a mouse model of intracerebral hemorrhage (Chen et al., 2020). DG is an important site for neurogenesis and plays a significant role in the function of the hippocampus, particularly in the formation of memory and spatial navigation (Anacker et al., 2018; Li et al., 2023). Consequently, it is an important research subject in the study of cognitive disorders (Le et al., 2014; Kong et al., 2024). However, our results showed that the colocalization correlation of TREM2 on astrocytes and neurons was not significant in DG, possibly due to distinct pathological changes between hemorrhagic and ischemic conditions.

TREM2 agonists and inhibitors of the PI3K/AKT signaling pathway are being explored as potential therapeutic strategies for neurological disorders (Zhang et al., 2019; Rodriguez et al., 2020; Singh et al., 2024). However, the clinical application of these drugs faces several challenges, including the need for precise molecular targeting, effective drug delivery systems, and the minimization of potential side effects. In our study, we selected EE as a non-pharmacological TREM2 agonist, with the goal of mitigating POCD by exposing mice with ischemic stroke to enriched environmental stimuli during the perioperative period. Our results align with previous studies in this regard (Yang et al., 2021). Cognitive neuroscience research suggests that cognitive processes can influence neural structures and functions. An enriched environment offers additional stimuli that promote neuronal growth and development. Concurrently, the brain’s adaptive capacity works to counteract increasing damage through the dynamics of neural networks (Gabriel et al., 2020; Yu and Wei, 2021; Di Castro et al., 2022).

Several limitations need to be mentioned in this study. Firstly, our assessment was confined to short-term neurofunctional outcomes in mice, necessitating further research to elucidate the long-term therapeutic effects of EE. Secondly, given that TREM2 expression was diminished by chronic ischemic stroke, it is imperative to investigate how acute ischemic stroke impacts TREM2 levels. Additionally, the activation and polarization state of microglia are pivotal in the progression of neural damage following ischemic stroke (Jiang et al., 2020). Our study did not focus on the differentiation of microglial subtypes; hence, relying solely on inflammatory cytokine results might not provide an accurate depiction of inflammation levels.

In this study, we initially established an ischemic stroke model by PT stroke to investigate the impact of EE on POCD. Our focus was on the potential neuroprotective role of TREM2 and its relationship with the PI3K/Akt signaling pathway. We observed that ischemic stroke resulted in neurological functional impairment, which was exacerbated by surgical trauma. However, exposure to EE could ameliorate these neurofunctional deficits. The improvement in neurofunctional impairment was associated with a reduction in inflammatory status and an upregulation of TREM2 in the hippocampus, along with the activation of the PI3K/AKT signaling pathway. We believe that our findings can contribute to the development of a safe perioperative prevention and treatment strategy for improving POCD in patients with stroke. Furthermore, our research may provide valuable insights for future investigations into the mechanisms underlying the combination of stroke and POCD.

## Data Availability

The datasets presented in this study can be found in online repositories. The names of the repository/repositories and accession number(s) can be found in the article/supplementary material.

## Supplementary material

Figure S1 The knockdown of TREM2 aggravated neuroinflammation in hippocampus of mice with ischemic stroke after surgery. **(A–E)** The concentrations of inflammation-associated factors in hippocampus at 24 h after surgery (*n* = 3). ns: no significant; **p* < 0.05, ****p* < 0.001, the SE+vehicle group vs. the SE +shRNA group; ##*p* < 0.01, ###*p* < 0.001, ####*p* < 0.0001, the EE+ vehicle group vs. the EE+ shRNA group. Error bars were represented as mean ± SD.
