# Supplementary figures and images for: Perioperative enriched environment attenuates postoperative cognitive dysfunction by upregulating microglia TREM2 via PI3K/Akt pathway in mouse model of ischemic stroke
Source: Front Neurosci. 2024 Dec 20;18:1520710. doi: 10.3389/fnins.2024.1520710 (PMC11695310; doi:10.3389/fnins.2024.1520710)

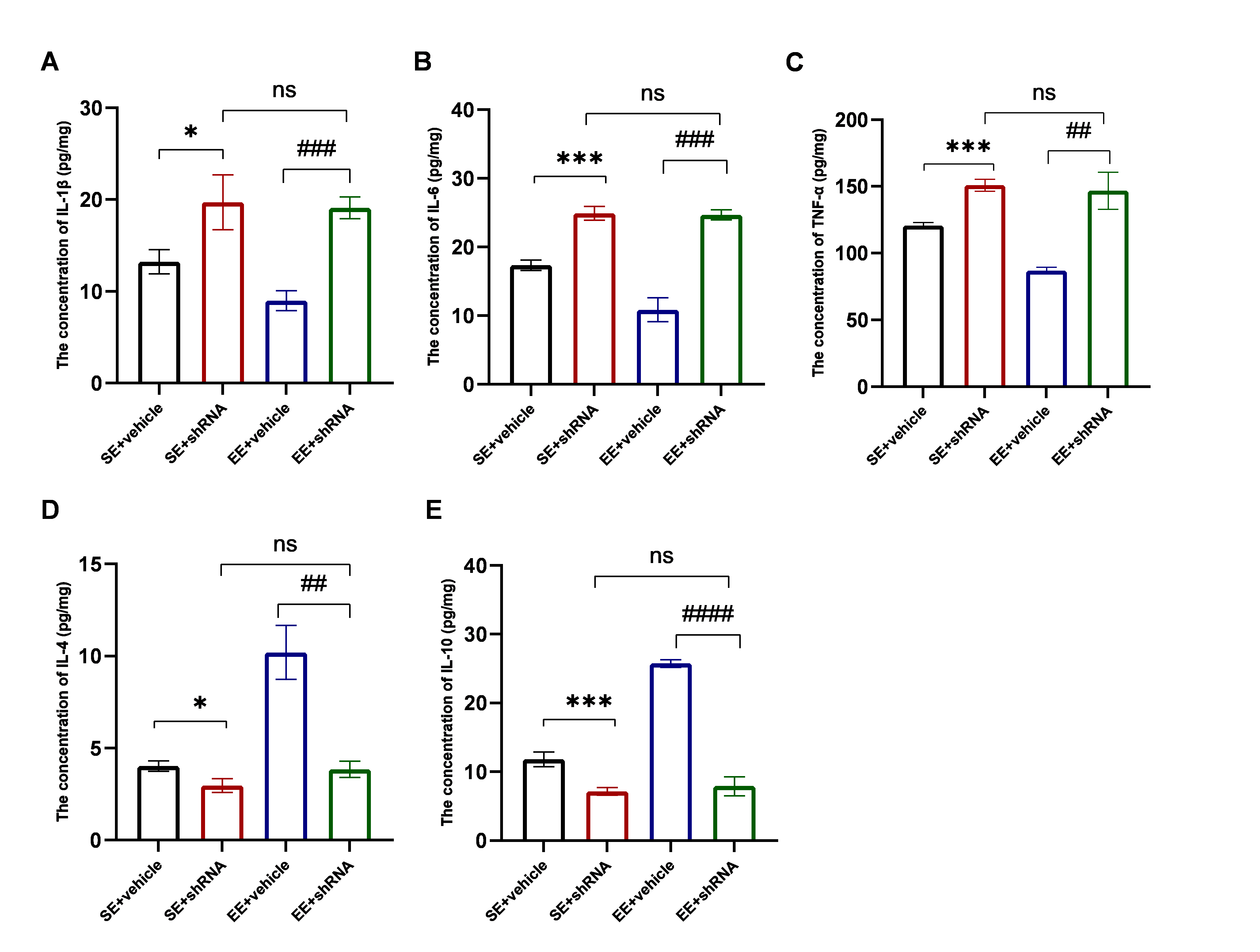

Supplement: Figure S1 — The knockdown of TREM2 aggravated neuroinflammation in hippocampus of mice with ischemic stroke after surgery. (A–E) The concentrations of inflammation-associated factors in hippocampus at 24 h after surgery (n = 3). ns: no significant; *p < 0.05, ***p < 0.001, the SE+vehicle group vs. the SE +shRNA group; ##p < 0.01, ###p < 0.001, ####p < 0.0001, the EE+ vehicle group vs. the EE+ shRNA group. Error bars were represented as mean ± SD. [file Image_1.tif]
